# A Synthetic Form of Frizzled 8-Associated Antiproliferative Factor Enhances p53 Stability through USP2a and MDM2

**DOI:** 10.1371/journal.pone.0050392

**Published:** 2012-12-06

**Authors:** Jayoung Kim, Susan K. Keay, Sungyong You, Massimo Loda, Michael R. Freeman

**Affiliations:** 1 Division of Cancer Biology and Therapeutics, Departments of Surgery and Biomedical Sciences, Samuel Oschin Comprehensive Cancer Institute, Cedars-Sinai Medical Center, Los Angeles, California, United States of America; 2 The Urological Diseases Research Center, Children’s Hospital Boston, Boston, Massachusetts, United States of America; 3 Departments of Surgery and Biological Chemistry and Molecular Pharmacology, Harvard Medical School, Boston, Massachusetts, United States of America; 4 Division of Infectious Diseases, Department of Medicine, the University of Maryland School of Medicine and VA Maryland Health Care Center, Baltimore, Maryland, United States of America; 5 Department of Medical Oncology, Harvard Medical School, Boston, Massachusetts, United States of America; 6 Center for Molecular Oncologic Pathology, Dana Farber Cancer Institute, Harvard Medical School, Boston, Massachusetts, United States of America; 7 Department of Pathology, Brigham and Women’s Hospital, Harvard Medical School, Boston, Massachusetts, United States of America; Vanderbilt University Medical Center, United States of America

## Abstract

Frizzled 8-associated Antiproliferative Factor (APF) is a sialoglycopeptide urinary biomarker of interstitial cystitis/painful bladder syndrome (IC/PBS), a chronic condition of unknown etiology with variable symptoms that generally include pelvic and/or perineal pain, urinary frequency, and urgency. We previously reported that native human APF suppresses the proliferation of normal bladder epithelial cells through a mechanism that involves increased levels of p53. The goal of this study was to delineate the regulatory mechanism whereby p53 expression is regulated by APF. Two APF-responsive cell lines (T24 bladder carcinoma cells and the immortalized human bladder epithelial cell line, TRT-HU1) were treated with asialo-APF (*as*-APF), a chemically synthesized form of APF. Biochemical analysis revealed that *as*-APF increased p53 levels in two ways: by decreasing ubiquitin specific protease 2a (USP2a) expression leading to enhanced ubiquitination of murine double minute 2 E3 ubiquitin ligase (MDM2), and by suppressing association of p53 with MDM2, thus impairing p53 ubiquitination. Biological responses to *as*-APF were suppressed by increased expression of wild type, but not mutant USP2a, which enhanced cell growth via upregulation of a cell cycle mediator, cyclin D1, at both transcription and protein levels. Consistent with this, gene silencing of USP2a with siRNA arrested cell proliferation. Our findings suggest that APF upregulates cellular p53 levels via functional attenuation of the USP2a-MDM2 pathway, resulting in p53 accumulation and growth arrest. These data also imply that targeting USP2a, MDM2, p53 and/or complex formation by these molecules may be relevant in the development of novel therapeutic approaches to IC/PBS.

## Introduction

More than one out of 77 people (3–8 million women and 1–4 million men) have been diagnosed with interstitial cystitis/painful bladder syndrome (IC/PBS) in the U.S. [1], [2], resulting in a great public health burden [3]–[5]. A specific diagnostic test is not yet available for IC/PBS, and inclusive and exclusive clinical diagnostic criteria listed in published NIDDK or AUA guidelines (e.g., results from urinalysis, urine culture, cystoscopy, biopsy of the bladder wall and urethra, and bladder distention) [6]–[10] are not consistently applied in general practice. Moreover, treatment of IC/PBS also remains challenging, as the use of a commonly prescribed IC/PBS medication, pentosan polysulfate (Elmiron), the first FDA-approved oral drug for this disease, results in symptom relief for only 30–60% of patients, and has side effects such as hair loss and gastrointestinal disturbance [1], [11]. Elucidation of the pathogenic mechanisms of IC/PBS is essential, as this may lead to the discovery of new approaches to therapy and to biomarkers for improved diagnosis[12]–[16].

Anti-proliferative factor (APF), a short sialoglycopeptide whose primary structure is 100% homologous to the putative sixth transmembrane domain of frizzled 8 (a Wnt receptor), has been proposed as a candidate biomarker for IC/PBS, based on the finding of significantly increased APF activity levels in urine from IC/PBS patients compared to normal controls [17], [18]. APF has been directly implicated in the pathogenesis of IC/PBS, as bladder epithelial cells from IC/PBS patients that produce this factor consistently proliferate at an abnormally slow rate compared to explanted epithelial cells from matched controls, and because purified human APF is a bladder epithelial cell growth inhibitor [15], [19]. Decreased cell proliferation along with reduced tight junction protein expression in response to APF is consistent with thinning, denudation and increased permeability observed in bladder epithelium of IC/PBS patients [20], [21].

APF signaling is mediated by the membrane receptor cytoskeleton-associated protein 4 (CKAP4), also known as CLIMP63, and requires S-acylation of CKAP4 by the palmitoyl acyltransferase DHHC2 [22]–[24]. The mechanism by which APF inhibits cell proliferation appears to involve downregulation of heparin binding EGF-like growth factor (HB-EGF) [25], with possibly related inhibition of AKT signaling [26]. APF also activates p38/MAPK signaling [27], [28] and upregulates p53 and p21 at both the protein and mRNA levels [26]. However, the specific mechanism by which APF regulates p53 levels in bladder epithelial cells is not clearly understood.

P53 is a tightly regulated transcription factor that arrests cell cycle transit or promotes cell senescence or apoptosis in response to stress stimuli. Activation of p53 results in accumulation of the protein in the nucleus and association with its functional partners [29]–[32]. Ubiquitination and degradation by the 26S proteasome are triggered by association of p53 with the RING-finger ubiquitin E3 ligase MDM2 [33]–[35]. This interaction is disrupted when cells detect DNA damage or other stresses, leading to stabilization and p53 activation. Overexpression of MDM2 is observed in many cancer types and correlates with aberrant p53 inactivation [32], [36], [37]; however, the role of MDM2 in bladder pathology is still poorly understood.

In this study we report findings of an investigation into the specific molecular mechanism whereby APF regulates p53 in bladder epithelial cells. Our results identify an important signaling network involving MDM2 and the de-ubiquitinase, USP2a, which is activated in response to chemically synthesized APF.

## Results

### Functionally Active Synthetic Asialo-APF (as-APF) Inhibits Proliferation by Regulation of p53

**Figure 1:**
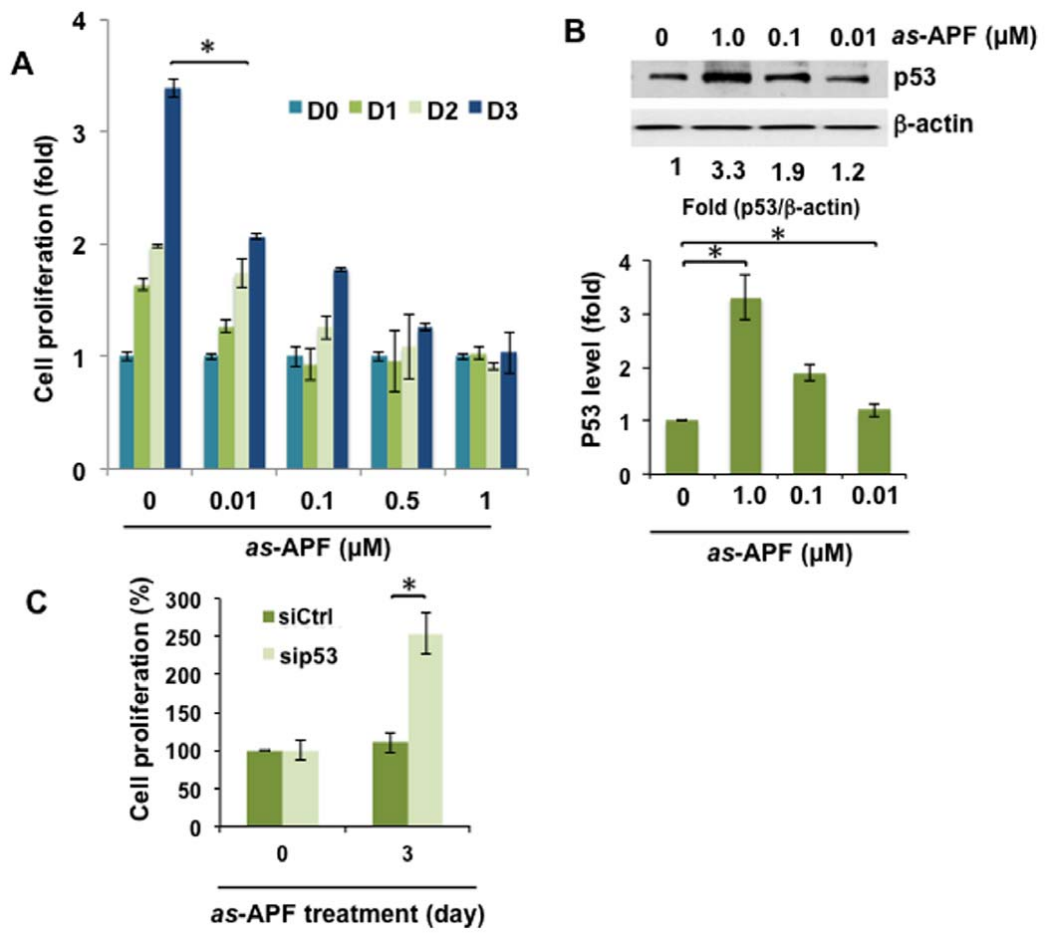
p53 is required for the growth inhibitory effect induced by the chemically synthesized *as*-APF. (**A**) Dose and time-dependent growth inhibitory effect of synthetic *as-*APF. T24 cells were incubated with varying doses of *as-*APF as indicated. Cell proliferation was determined by crystal violet assay at 0, 1, and 3 days after treatment. Experiments were performed in triplicate. (**B**) *as-*APF increases p53 expression. T24 cells were incubated *as-*APF (0, 0.01, 0.1 or 1 µM) containing serum free medium for 3 days. Cell lysates were prepared for immunoblot analysis using antibodies against p53 or β-actin. Fold changes of p53/β-actin are shown in the graph. (**C**) Gene silencing of p53 recovers growth arrest by *as-*APF. T24 cells were transiently transfected using siRNAs for p53 (sip53) or control (siCtrl). 24 h after transfection, cells were serum starved for 16 h and incubated with *as-*APF containing medium (1 µM) for additional 3 days. Data are expressed as mean±SD. *p<0.05 (Student’s t-test).

To understand the mechanism whereby p53 levels are increased in response to APF in bladder epithelial cells, asialylated-APF (*as*-APF) (2-3Galβ1-3GalNAcα-*O*-TVPAAVVVA), and its inactive nonglycosylated negative control peptide (TVPAAVVVA) were used in this study [38]. Consistent with the inhibition of cell proliferation seen in responsive to native APF in T24 bladder cells as determined by crystal violet staining, 3 days of treatment with synthetic *as*-APF increased p53 protein levels in a concentration-dependent manner (Fig. 1A). 1 µM *as-*APF significantly increased p53 level about 3.3 fold (Fig. 1B), suggesting p53 is involved in the inhibitory effect of *as-*APF on cell proliferation. In comparison, the control mock peptide had no effect on p53 levels (not shown). To assess the involvement of p53 in growth suppression by *as*-APF, RNA interference experiments were performed using p53 siRNA duplexes. Knockdown of p53 diminished the inhibitory effect of synthetic *as*-APF on cell growth (Fig. 1C), consistent with previous observations using native HPLC-purified APF [39]. Combined with previous observations on the biological effects of *as-*APF [15], [26], [38], our results indicate that *as-*APF has the same activity as the HPLC-purified native APF peptide from the spent cell medium of bladder explants from patients who were diagnosed with IC/PBS [15], [21], [23], [25].

### as-APF Enhances p53 Stability via Suppression of MDM2

**Figure 2:**
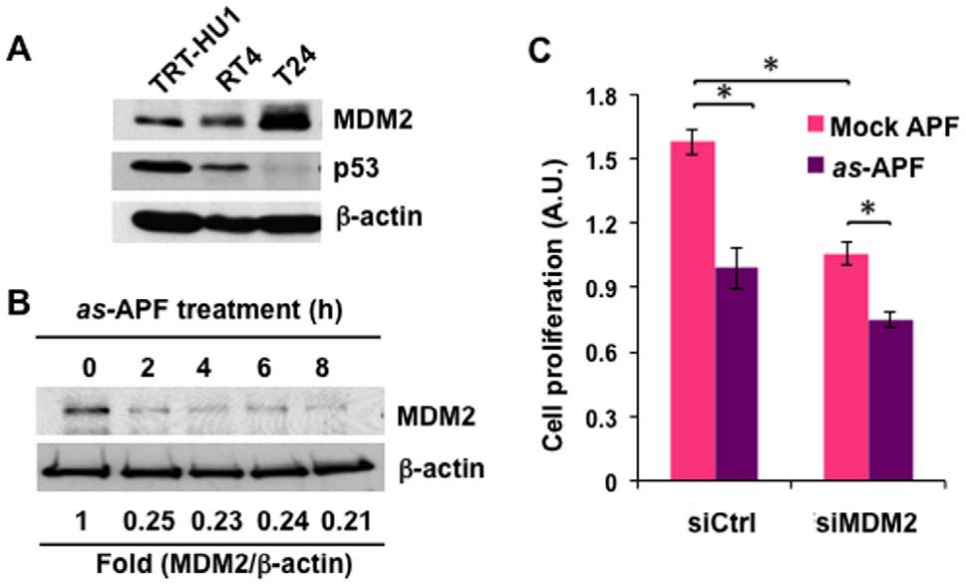
*as-*APF downregulates MDM2, leading to inhibition of cell proliferation. (**A**) Inverse expression pattern of p53 and MDM2 in bladder cell lines. Protein extracts were prepared from growing TRT-HU1, RT4 and T24 bladder cell lines for western blot analysis using antibodies against MDM2, p53 and β-actin. (**B**) MDM2, an E3 ubiquitin ligase, is decreased in response to *as-*APF. T24 cells were treated with APF (1 µM) for the indicated times (1, 2, 4, 6 or 8 h). Whole cell lysates were prepared for immnuoblot with anti-MDM2 antibody. Fold changes compared to time 0 were calculated based on band intensities after normalization to β-actin. (**C**) Silencing of MDM2 decreases cell proliferation with or without *as-*APF treatment. Transfected T24 cells with siCtrl or siMDM2 were incubated in 1 µM *as-*APF (or negative control peptide)-containing medium for 3 days. Cell proliferation was determined by crystal violet assay. *p<0.05 (Student’s *t-*test).

We next sought to understand the molecular mechanism whereby the p53 expression level elicits growth arrest in response to *as*-APF. We observed an apparent inverse relationship between p53 expression and expression of its negative regulator, MDM2, in the TRT-HU1 immortalized bladder epithelial cell line, recently developed by us [40], and in T24 bladder cancer cells (Fig. 2A). These data suggest that p53 accumulation in response to *as*-APF may involve MDM2, and that *as-*APF may block p53 ubiquitination and protein degradation as part of the mechanism by which it increases p53 protein levels [39]. Time course western blot analysis showed that *as*-APF treatment rapidly decreased MDM2 protein at 2 h (∼75% reduction) (Fig. 2B). In experiments where T24 cells were treated with *as*-APF, we noted that MDM2 gene silencing itself suppressed proliferation, and there was only a mild additive effect of *as*-APF in the setting of MDM2 knockdown (Fig. 2C). These data imply that *as*-APF inhibits T24 bladder cell proliferation mainly through the MDM2 and p53 axis.

### as-APF Suppresses p53 Ubiquitination and Cell Proliferation

**Figure 3:**
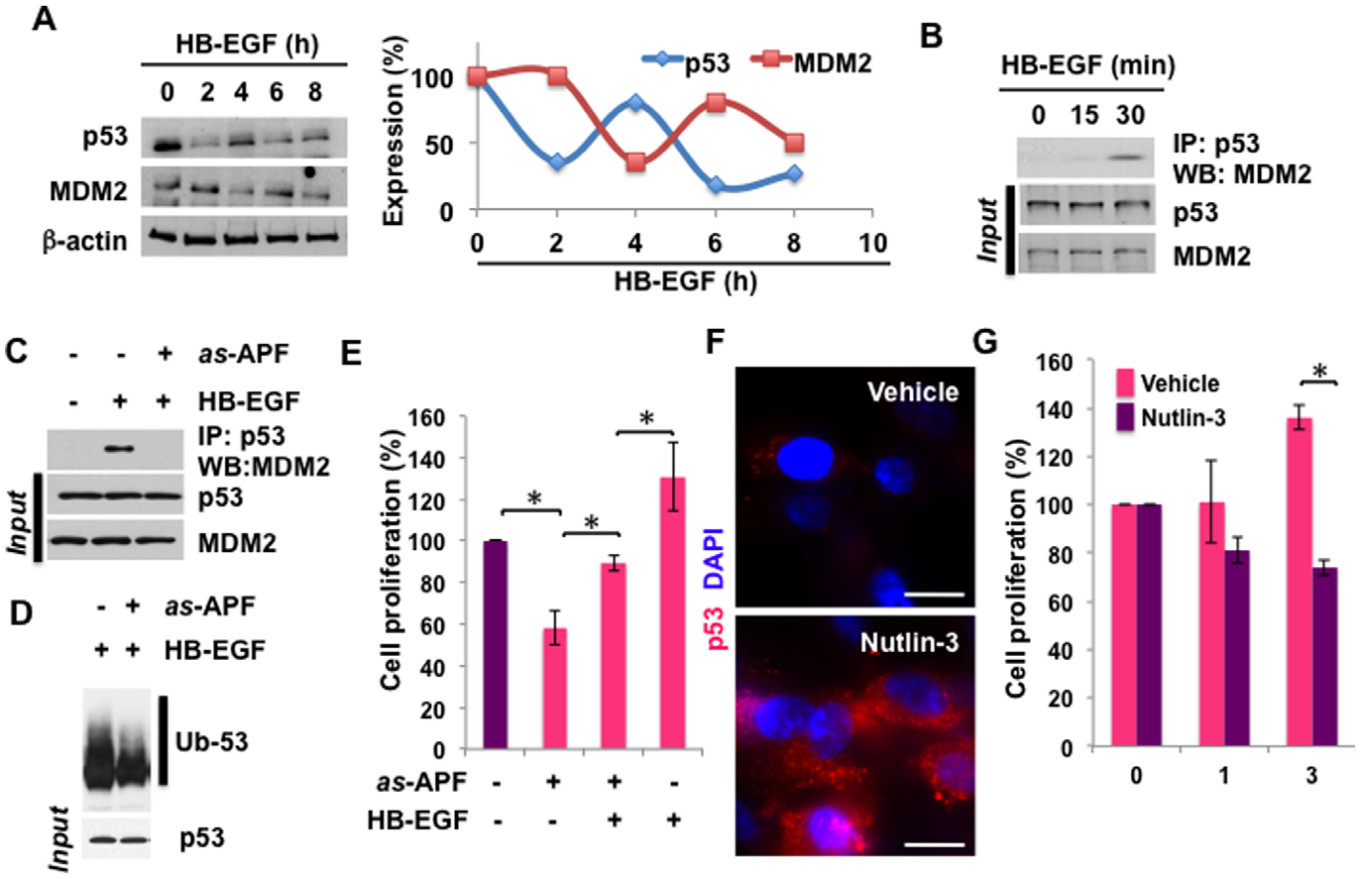
*as-*APF treatment antagonizes HB-EGF by regulation of MDM2 and p53. (**A**) MDM2 expression is negatively correlated with p53 level. T24 cells were serum starved for 16 h and stimulated with 10 ng/ml HB-EGF containing serum free medium. At 0, 2, 4, 6, and 8 h after treatment cells were harvested and whole cell lysates were prepared for immunoblot analysis. Protein expression levels of MDM2 or p53 are shown normalized to β-actin (right). (**B**) Association of p53 and MDM2. T24 cells were harvested after HB-EGF treatment for 0, 15 or 30 min for immunoprecipitation (IP) with anti-p53 antibody. Western blot was then done with anti-MDM2 antibody to determine the binding of p53 and MDM2 in response to HB-EGF. (**C**) *as-*APF reverses association of p53 and MDM2 induced by HB-EGF. T24 cells were incubated in *as-*APF (1 µM) and/or HB-EGF (10 ng/ml) containing medium for 30 min. Whole cell lysates were subjected to co-IP and western blot analysis using antibodies against p53 and MDM2. (**D**) *as-*APF alleviates the HB-EGF-induced p53 ubiquitination. Cells were stimulated with *as-*APF (1 µM) and/or HB-EGF (10 ng/ml) in the presence of MG132 (10 µM) for 30 min. Whole cell lysates were used for IP with anti-p53 antibody and following immunoblot analysis with anti-p53 antibody. (**E**) Antagonistic effects of *as-*APF and HB-EGF on cell growth. T24 cells were incubated with *as-*APF (1 µM) and/or HB-EGF (10 ng/ml) containing medium for 3 days and proliferation was determined by crystal violet assay. *p<0.05. (**F**) Inhibition of association between MDM2 and p53 by Nutlin-3 increases p53 expression. T24 cells were incubated in 10 µM Nutlin-3- or Vehicle-containing medium for 3 days. Immunofluorescence staining showed that p53 level was increased by Nutlin-3. p53 appears red. Scale bar represents 10 µm. (**G**) Nutlin-3 decreases cell proliferation. T24 cells were incubated in 10 µM Nutlin-3- or Vehicle-containing medium for the indicated times (0, 1, and 3 days). Cell proliferation was determined by crystal violet assay. *p<0.05.

Consistent with the conclusion that p53 is regulated by MDM2, which is downregulated in response to *as-*APF treatment, p53 protein levels fluctuated inversely and time dependently with MDM2 in response to HB-EGF, a potent pro-proliferative growth factor for bladder epithelial cells and an APF antagonist [27] (Fig. 3A). Given this, we tested the association of p53/MDM2 in response to HB-EGF [27]. Immunoprecipitation (IP) and western blot analysis demonstrated that HB-EGF stimulated p53/MDM2 complex formation (Fig. 3B). Given the opposing responses of MDM2 and p53 elicited by HB-EGF (Fig. 3A), the enhanced association of p53 and MDM2 by HB-EGF stimulation (Fig. 3B) supported the hypothesis that MDM2 may negatively regulate p53 expression, possibly by stimulating direct binding of MDM2 to p53 following HB-EGF treatment [33]–[35]. Association of p53 and MDM2 by HB-EGF was completely inhibited by *as-*APF (Fig. 3C), and the level of p53 ubiquitination was suppressed by *as-*APF pre-treatment (Fig. 3D). These findings suggest that *as-*APF inhibits MDM2 binding to p53, and leads to p53 accumulation. Furthermore, HB-EGF significantly abrogated the effect of *as-*APF on cell growth, while *as-*APF blocked the enhancement of cell proliferation by HB-EGF (Fig. 3E). Levels of HB-EGF were shown to be lower in urine from IC/PBS patients in comparison to normal controls, and HB-EGF levels were shown to increase, while APF activity levels decreased, after cystoscopic hydrodistention, a common clinical procedure to relieve IC symptoms [41]. HB-EGF abrogated the effects of APF on human bladder epithelial cells [25], and antagonized native APF-stimulated p38MAPK signaling and cell cycle arrest [27], [28]. Nutlin-3, a small-molecule inhibitor of the p53-MDM2 interaction, upregulated p53 levels in the T24 bladder cells (Fig. 3F) and resulted in growth suppression (Fig. 3G). Taken together, these data suggest that *as*-APF reduces complex formation between p53 and MDM2, leading to p53 accumulation and cell growth inhibition.

### as-APF Regulates Expression of the USP2a Deubiquitinase and Stability of MDM2

**Figure 4:**
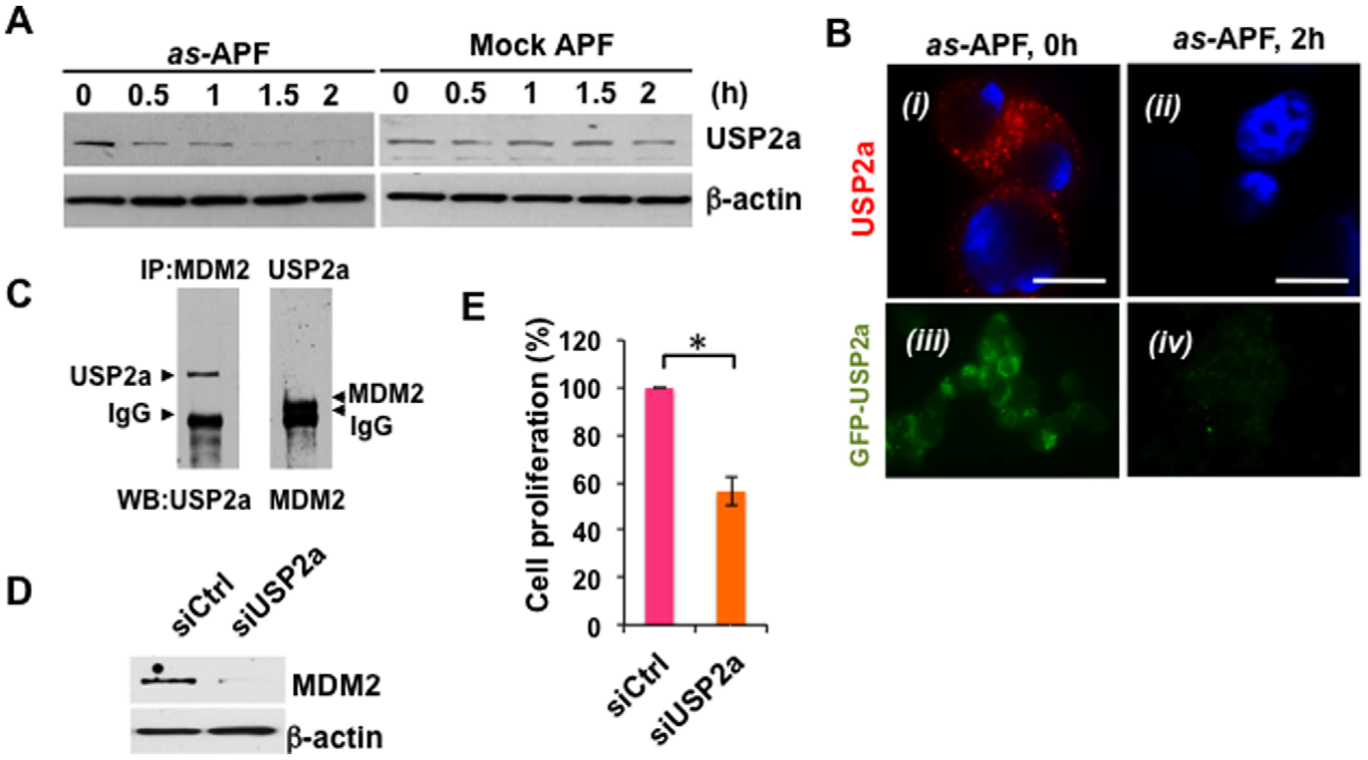
Regulation of USP2a and MDM2 in response to *as-*APF. (A ) *as-*APF downregulates USP2a expression (immunoblot). T24 cells were incubated with 1 µM *as*-APF or control peptides for the indicated times (0, 0.5, 1, 1.5, or 2 h). Western blot with anti-USP2a antibody showed that the level of USP2a level was stably decreased in response to *as*-APF treatment, but this was only transiently reduced by control mock APF peptide. (**B**) Reduced USP2a by *as*-APF treatment (IF staining). (*i* and *ii*) After incubation with 1 µM *as-*APF for 2 h, cells were fixed and stained with anti-USP2a antibody and a Cy3-conjugated secondary antibody (red). Nuclei were counterstained with DAPI. Scale bar represents 10 µm. (*iii* and *iv*) USP2a expression in T24 cells transfected with a GFP-USP2a construct was diminished in response to APF treatment. Green, USP2a. (**C**) Association of MDM2 and USP2a. Exponentially growing T24 cells were used for co-IP and western blot analysis with antibodies against USP2a and MDM2. (**D**) Knockdown of USP2a results in reduced MDM2 expression. T24 cells were transiently transfected with USP2a siRNA (siUSP2a) or siCtrl, and whole cell lysates were used for western blot analysis. (**E**) Gene silencing of USP2a inhibits cell proliferation. 3 days after transient transfection with siRNA of USP2a, cell proliferation was measured by crystal violet.

The above findings raised the question of how MDM2 is regulated in response to *as-*APF. A previous report by Stevenson et al. demonstrated that MDM2 is a target of the ubiquitin specific protease 2a (USP2a), a de-ubiquitinating enzyme [42]. Overexpression of USP2a promoted accumulation of MDM2 by reversing ubiquitination and subsequent protein degradation, while reduced USP2a destabilized MDM2 and caused accumulation of p53 protein [42]. We decided to assess whether USP2a affects p53 expression by modulating MDM2 stability in response to *as-*APF. Time course western blot analysis demonstrated that *as-*APF treatment rapidly decreased USP2a level within 30 min, while the control peptide had no effect (Fig. 4A). Immunofluorescence imaging of T24 cells treated with control (mock APF) or *as-*APF peptides confirmed that USP2a (punctuate pattern, in red) rapidly diminished and no USP2a was observed 2 h after treatment with 1 µM *as-*APF (Fig. 4B, *i* and *ii*). USP2a-GFP level (in green) was reduced in response to APF treatment in T24 cells transiently transfected with a USP2a-GFP expression construct (Fig. 4B, *iii* and *iv*). In addition, in untreated T24 bladder cells, MDM2 and USP2a (Fig. 4C), but not p53 and USP2a (data not shown), formed a complex. These findings suggest the possibility that USP2a plays a role in regulation of MDM2 and that USP2a may stabilize MDM2 by competing with ubiquitin for binding to MDM2, as suggested in the Stevenson et al. report [42]. Indeed, when USP2a was silenced by siRNA, MDM2 protein levels and proliferation were significantly reduced in *as*-APF-treated cells (Fig. 4D and 4E), implying that USP2a is an important mediator of MDM2 activity regulating bladder cell proliferation.

### Enforced USP2a Reverses the Anti-proliferative Effect of as-APF

**Figure 5:**
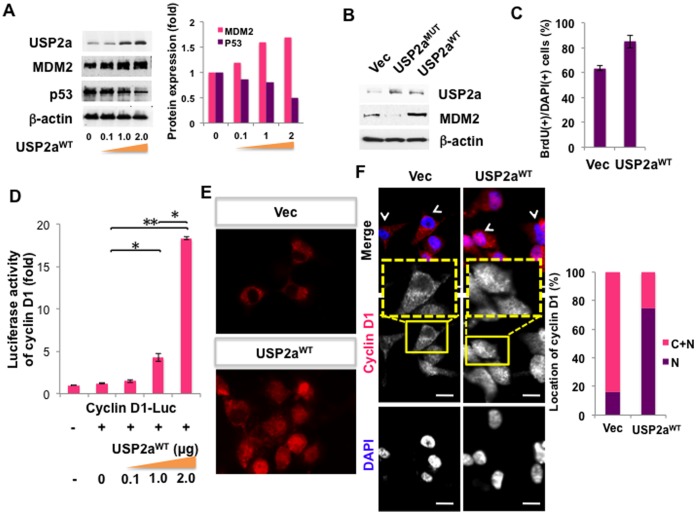
Catalytically active USP2a, USP2a^WT^, alters MDM2 and cyclin D1 expression. (**A**) Enforced expression of wild type USP2a (USP2a^WT^) enhances MDM2 level and decreases p53 expression (western blot). T24 cells were transfected with varying doses of USP2a^WT^ plasmid. 2 days later, whole cell lysates were prepared and subjected to western blot with MDM2 or p53 antibodies. Experiments were performed 3 times and blot images are representative. Fold change was calculated based on band intensities and normalized to β-actin (right graph). (**B**) USP2a^WT^, but not catalytically inactive USP2a^MUT^, enhances MDM2 expression (Western blot). T24 cells were transfected with Vector, USP2a^WT^ or USP2a^MUT^ constructs, and prepared for western blot. Data are representative of at least 3 experiments. (**C**) Proliferation is enhanced by overexpression of USP2a^WT^ (BrdU staining). 3 days after transfection with USP2a^WT^ construct (or Vector control), T24 cells were stained with BrdU staining reagent for visualization of proliferating cells. Green indicates proliferating cells. Nuclei were stained with DAPI (Blue). (**D**) Cyclin D1 transcription is induced by USP2a^WT^ (promoter assay). Cyclin D1 luciferase promoter construct, MDM2 and varying doses of USP2a^WT^ (0, 0.1, 1 or 2 µg) were transfected into T24 cells. Three days after transfection, cyclin D1 promoter activity was measured by luciferase assay. *p<0.05. **p<0.005. (**E–F**) Cyclin D1 protein expression and nuclear localization are increased by USP2a^WT^ overexpression (IF imaging). Immunofluorescence staining for cyclin D1 in T24 cells was performed 2 days after overexpression of USP2a^WT^. Cells were fixed and stained with specific antibody to cyclin D1 (red) and mounted with DAPI (blue) solution. Scale bar represents 10 µm. (**F**) Localization of cyclin D1 was quantified. N, only nuclear resident cyclin D1; C+N, localized in nucleus as well as cytoplasmic compartments.

We further expanded our findings suggesting a role for USP2a-MDM2-p53 signaling in APF-mediated inhibition of cell growth. Enforced expression of wild type USP2a (USP2a^WT^) increased MDM2 and decreased p53 levels (Fig. 5A and 5B). In comparison, a catalytically inactive USP2a mutant, (USP2a^MUT^) did not promote enhanced MDM2 expression (Fig. 5C), suggesting that USP2a enzyme activity is critically required for regulation of the MDM2-p53 signaling pathway that responds to APF stimulation. Bromodeoxyuridine (BrdU) staining, which allows detection of proliferating cells in S phase, demonstrated that USP2a^WT^ enhanced proliferation (Fig. 5C). We assessed the mechanism by which USP2a alters cell proliferation using a cyclin D1 luciferase promoter-reporter as a read-out because cyclin D1 has been previously identified as a USP2a target as well as an important cell cycle regulator in bladder cells [43]. USP2a activated the cyclin D1 promoter in a concentration dependent manner (Fig. 5D). USP2a also enhanced cyclin D1 protein expression and nuclear localization (Fig. 5E and 5F), suggesting that USP2a plays a role in proliferation by using cyclin D1 as a mediator. These data also imply that complex formation between USP2a/MDM2 may be required for MDM2 stabilization, leading to degradation of p53.

**Figure 6:**
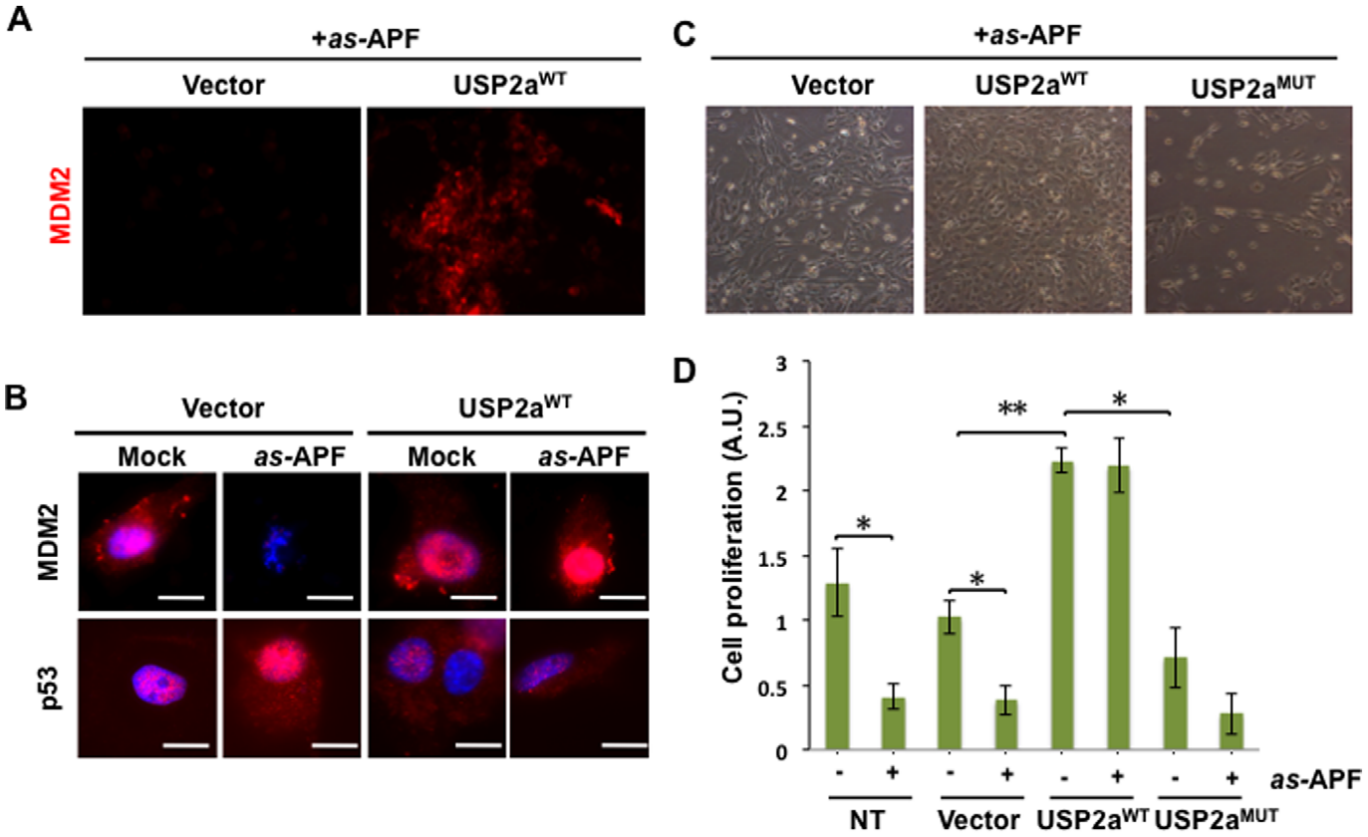
USP2a^WT^ blocks the growth inhibitory effect of *as-*APF. (**A–B**) USP2a^WT^ abrogated *as-*APF’s effect on MDM2 and p53 levels (IF imaging). MDM2 or p53 were labeled with specific primary antibodies and Cy3.5 conjugated secondary antibody (red). Nuclei were stained with DAPI (Blue). (**C–D**) Enforced expression of USP2a^WT^ alleviates the *as-*APF-induced growth inhibition. T24 cells were transiently transfected with vector, USP2a^WT^ or USP2a^MUT^ constructs. One day after transfection, cells were incubated in the 1 µM *as-*APF containing serum free medium for an additional 3 days. Proliferation was measured by crystal violet assay and representative cell images are shown in C.

Based on these results, we thus hypothesized that reduced USP2a elicited by *as-*APF may allow MDM2 degradation, resulting in a subsequent increase in p53-mediated cell growth inhibition. To test this hypothesis, we examined the effects of enforced USP2a expression in the presence or absence of *as-*APF. Immunofluorescence microscopy demonstrated that USP2a^WT^ substantially sustained MDM2 levels in the presence of *as-*APF, while control cells (Vector) showed almost no MDM2 expression (Fig. 6B, upper panels). P53 expression was increased in response to *as-* APF (Fig. 6B, left lower panels). However, when USP2a^WT^ was overexpressed, p53 level was greatly reduced in response to APF (Fig. 6B, right low panels), suggesting that enforced USP2a expression impaired the effect of *as-*APF on MDM2 and p53. To assess the effect of altered expression of USP2a, we analyzed cell proliferation after transfection of T24 cells with USP2a^WT^ or USP2a^MUT^ constructs. Compared to controls, USP2a^WT^ cells were more proliferative in the absence or presence of *as-*APF, while USP2a^MUT^ had no effect (Fig. 6C and 6D). No growth suppression was observed in response to *as-*APF when USP2a^WT^ was overexpressed, suggesting that active USP2a reverses the APF inhibitory effect on proliferation; in comparison, USP2a^MUT^ did not affect cell proliferation or the effects of APF (Fig. 6D).

### as-APF Activates the USP2a-MDM2-p53 Network in Human Non-malignant Bladder Epithelial Cells

**Figure 7:**
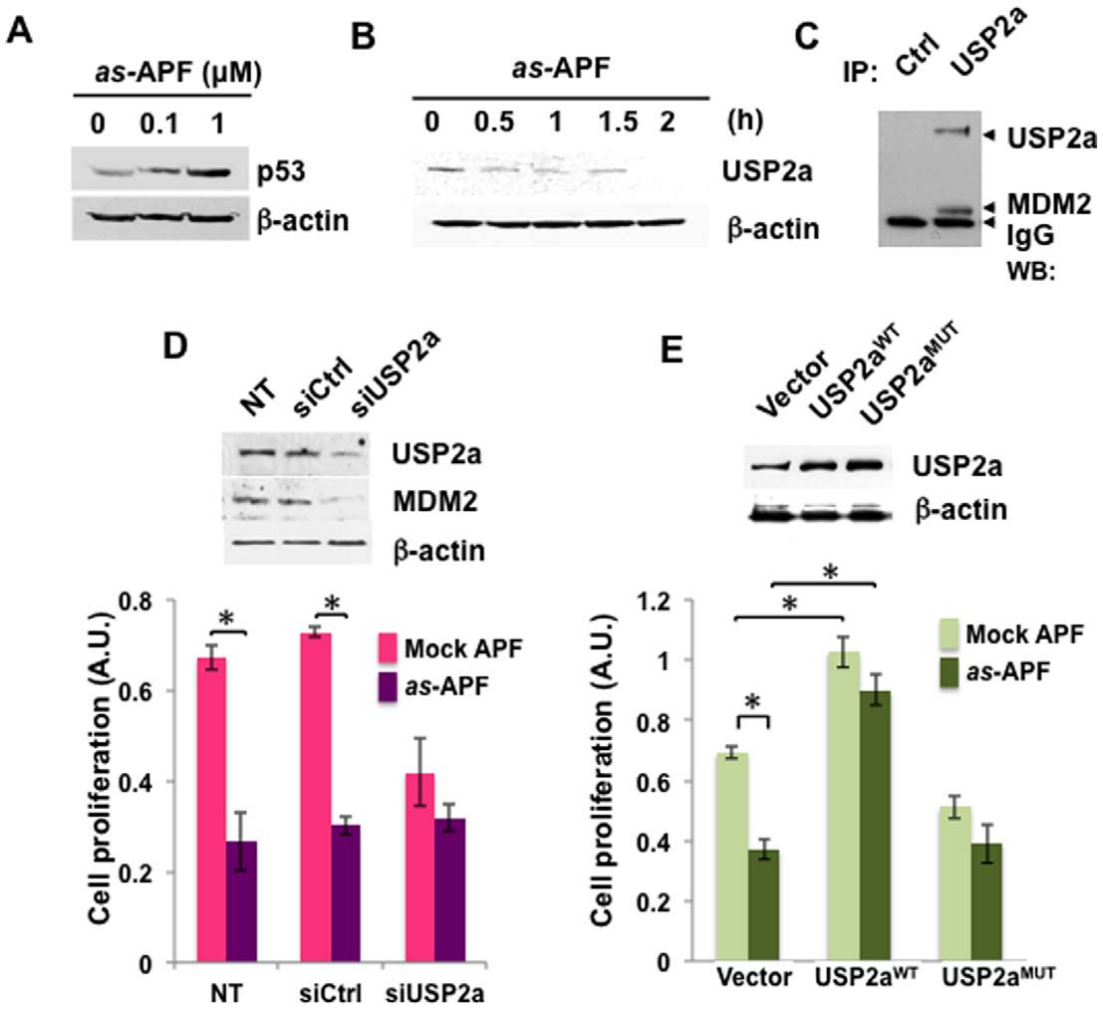
*as-*APF increases p53 expression by modulating USP2a and MDM2 in TRT-HU1, immortalized human normal bladder epithelial cells. (**A**) p53 levels were enhanced by *as-*APF treatment. TRT-HU1 cells were incubated in *as-*APF-containing serum free medium for 3 days before harvesting cells for western blot analysis with anti-p53 antibody. (**B**) Endogenous USP2a level is rapidly decreased in response to *as*-APF treatment in TRT-HU1 cells. (**C**) Association of USP2a and MDM2 in TRT-HU1 cells. IP was performed using whole cell lysates from TRT-HU1 cells in exponential growth with anti-USP2a antibody, followed by western blot analysis with antibodies against USP2a and MDM2. Normal mouse IgG was used as an IP control (Ctrl). (**D**) USP2a loss decreases MDM2 and cell proliferation. TRT-HU1 cells were transiently transfected with siRNA of USP2a (or siCtrl). One day after transfection, cells were treated with 1 µM *as-*APF (or mock peptide)-containing medium. Three days later, cells were harvested for western blot analysis and proliferation assay. *p<0.05, NT, non-transfected cells. (**E**) Proliferation is enhanced by overexpression of USP2a^WT^, but not by USP2a^MUT^. Cells transfected with USP2a^WT^, USP2a^MUT^ or vector control (Vec) constructs were treated with 1 µM *as-*APF or mock peptide for 3 days. Western blot and proliferation assay were performed as above. Data are represented as mean +/− SD. *p<0.05.

**Figure 8:**
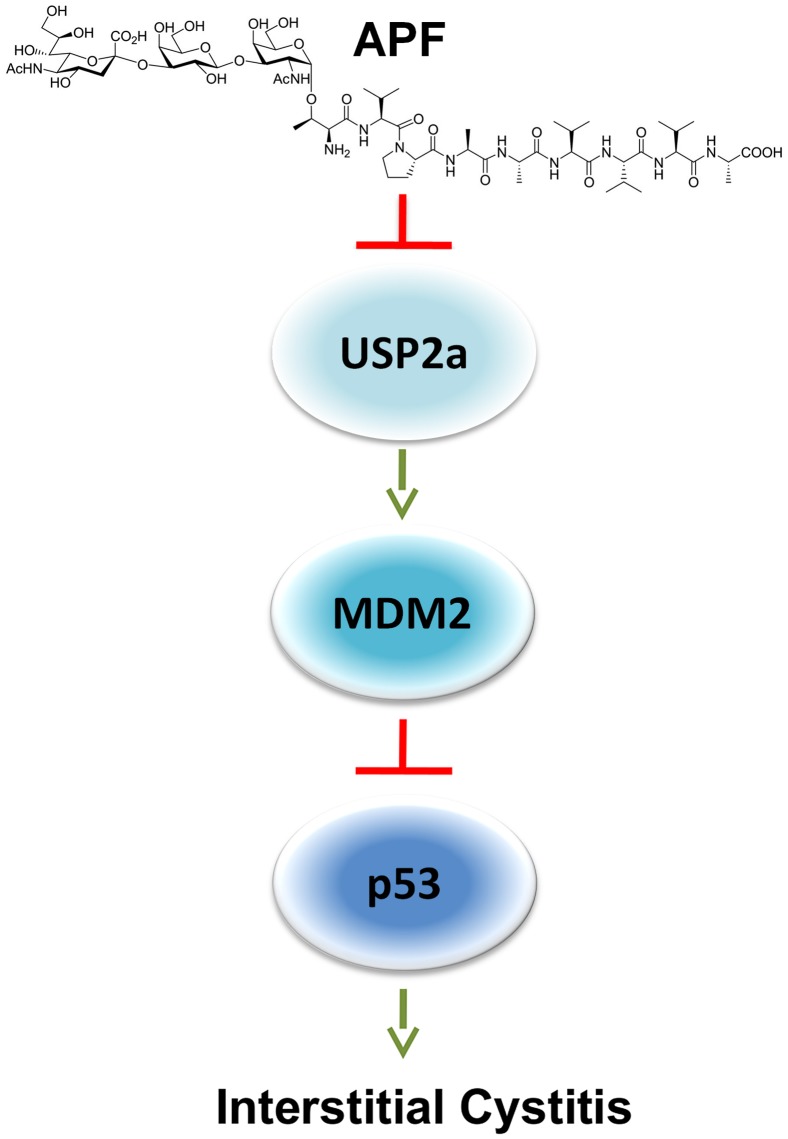
Diagram proposing the points at which the USP2a-MDM2-p53 network mediates the effect of APF on urothelial cell proliferation.

To further examine the regulatory role of the USP2a-MDM2-p53 network in APF-induced growth arrest, we performed additional experiments using TRT-HU1 cells [40]. *as-*APF at 1 µM markedly increased levels of p53 and rapidly diminished USP2a levels over 3 days in this cell background (Fig. 7A and 7B). A direct association between USP2a and MDM2 was shown by IP and western blot in untreated cells (Fig. 7C). Knockdown of USP2a by siRNA resulted in a decrease in MDM2 level as well as inhibition of growth in the presence of *as-*APF (Fig. 7D). Enforced expression of USP2a^WT^, but not USP2a^MUT^, abrogated the growth inhibition seen following *as*-APF treatment (Fig. 7E). Taken together, these results suggest that USP2a-MDM2-p53 is a signaling axis that mediates the physiologic effects of APF in bladder epithelial cells. A diagram of the USP2a-MDM2-p53 signaling network that is engaged in response to APF is shown in Fig. 8.

## Discussion

Despite growing clinical interest in IC/PBS, a symptom-based bladder disease that causes chronic pain, increased frequency, and urgency, the molecular basis of IC/PBS remains uncharacterized. Because IC/PBS symptoms overlap with other common gynecologic and urologic conditions (such as pelvic inflammatory disease, urethritis, cystitis, and prostatitis), specific and unique diagnostic markers are urgently needed.

We previously reported that the p53 signaling network is activated by APF, a urine IC/PBS glycopeptide that produces effects in primary normal bladder epithelial cells that resemble changes seen in IC/PBS cell explants in vitro as well as changes seen in the bladder of IC/PBS patient biopsies [20], [39]. In this study, we sought to gain further insight into the mechanism by which APF increased p53 levels in bladder epithelial cells. We employed two new reagents in this study: (1) a synthetic form of APF (*as-*APF), and (2) an immortalized, benign, and APF-responsive bladder cell line that we recently developed [40]. Our work defines a new mechanism of APF-mediated signaling, in which a molecular network involving USP2a, MDM2, and p53, is activated in bladder epithelial cells in response to *as*-APF. Our findings support the following conclusions: (1) synthetic *as-*APF decreases USP2a and MDM2 levels, (2) *as*-APF blocks a direct association between p53 and MDM2, resulting in decreased p53 ubiquitination and protein degradation, and (3) the effect of *as*-APF on bladder epithelial cell proliferation can be blocked by enforced expression of USP2a.

USP2a was previously shown to be a regulator of the MDM2/p53 pathway in a range of tumor cells, including oral squamous cell carcinoma, testicular embryonal carcinoma, prostate carcinoma, and breast carcinoma [44]–[46]. USP2a, which forms a complex with MDM2 [42], the MDM2 homologue MDMX [47], [48], FASN (fatty acid synthase) [49], cyclin D1 [50] and Aurora A [51], is positively linked to tumor progression [52]. Downregulation of USP2a accelerates ubiquitin-dependent degradation of proteins such as MDM2, FASN and EGFR [42], [47], [49], [50]. However, a role for USP2a has not been established in any bladder diseases, including bladder cancer and IC/PBS. Our findings suggest that the altered ubiquitination status triggered by APF, a bioactive peptide, results in the impaired regulation of key proteins during pathological conditions in the bladder.

Previous studies using native APF, which was HPLC-purified from human bladder epithelial cells [39], demonstrated that p53 plays a critical role in APF-induced inhibition of cell proliferation. Synthetic *as*-APF was also recently shown to increase p53 mRNA and protein expression in T24 bladder carcinoma cells [26]. Our results here suggest that USP2a and MDM2 are additional components of the p53 network as upstream regulators of p53 in response to *as*-APF-stimulated signal activation in bladder cells.

In addition to altered p53 signaling, several interesting other signaling abnormalities have been observed in cell explants from patients with IC/PBS and APF-stimulated bladder cells [28]. For example, in APF-responsive T24 cells, MAPK signaling was also shown to be involved in APF-mediated cell growth arrest [27]. APF rapidly and transiently activates p38MAPK, which antagonizes Erk/MAPK signaling [27]. APF has also been shown to inhibit Akt phosphorylation and increase downstream-specific phosphorylation of β-catenin [26]. APF-responsive cells further exhibit altered expression of genes known to play a role in tight junctions, cellular proliferation and tumorigenesis (e.g. E-cadherin, vimentin, and zonula occludens protein-1) [21]. Very recently, a quantitative proteomics study performed by our group identified a global network of APF-regulated proteins, within which β-catenin is a key node [53]. Expression levels of β-catenin and a downstream target, cyclooxygenase-2, were altered in epithelial cells explanted from IC bladder biopsies compared with control tissues [53]. However, the relationship of the multiple signaling pathways affected by APF to each other remains to be determined, and the relationship of any of these pathways to the characterized APF receptor CKAP4 is also not fully understood.

Our results have provided evidence that APF perturbs protein stability through a balance of ubiquitination and deubiquitination arising from the action of the UPS2a/MDM2/p53 network. Our biochemical and functional analyses suggest that the USP2a-MDM2-p53 axis may be pertinent to pathologic changes in the bladder wall associated with IC/PBS. To our knowledge, this is the first evidence implicating USP2a and MDM2 in biological effects induced by APF. These findings therefore expand our understanding of the mechanism whereby APF inhibits bladder epithelial cell proliferation, and suggest that components of this mechanism might serve as useful clinical features, diagnostic indicators or as novel therapeutic targets. In particular, a strategy targeting USP2a degradation or complex formation between USP2a/MDM2/p53 may be fruitful in the development of therapeutic approaches to IC/PBS.

## Experimental Procedures

### Reagents

*as-*APF and nonglycosylated nagative control peptides were purchased from PolyPeptide Laboratories, Inc. (San Diego, CA), and they were synthesized according to previously described methods [15], [26], [38]. Heat inactivated and dialyzed fetal bovine serum (FBS) and Lipofectamine 2000 were purchased from Invitrogen (Carlsbad, CA). Micro BCA protein assay kit and protease inhibitor cocktail tablets were obtained from Pierce (Rockford, IL) and Roche Diagnostics (Basel, Switzerland). Premade SDS-PAGE (4–20%) gels, Coomassie Blue R-250 staining solution and destaining solution were from Bio-Rad (Hercules, CA). Small interfering RNAs (siRNAs) and OFF-TARGET controls were from Dharmacon (Chicago, IL). 4′,6-diamidino-2-phenylindole (DAPI) was purchased from Vector Laboratories (Burlingame, CA). Antibodies against MDM2, p53, USP2a, cyclin D1 and β-actin, were purchased from Abcam (Cambridge, MA), CalBiochem (San Diego, CA), ABGENT (San Diego, CA) and Cell Signaling Technology (Danvers, MA), respectively. All other reagents were obtained from Sigma-Aldrich or Promega (Madison, WI).

### Cell Culture

APF-responsive human bladder cancer T24 cells, or RT4 cells were obtained from the American Type Culture Collection (ATCC). Cells were cultured in McCoy’s 5A medium containing 10% FBS, 2 mM L-glutamine, 100 units/mL penicillin, and 100 µg/mL streptomycin at 37°C in a humidified incubator with 5% CO_2_. Immortalized human normal bladder epithelial TRT-HU1 cells were developed and maintained as described [40]. For transient transfection, cells were grown to ∼80% confluence in 100 mm dishes, at which time they were transiently transfected with plasmid constructs containing cDNAs of MDM2, USP2a^WT^ or catalytically inactive USP2a (USP2a^MUT^), which were cloned into pEGFP and used for transfection. To silence protein expression, cells were transfected with 80 nmol/L of small interfering RNAs (siRNAs) against p53, MDM2 or USP2a (Dharmacon, Lafayette, CO) using Lipofectamine 2000 (Invitrogen, Carlsbad, CA) according to the manufacturer’s instructions. OFF-TARGET siRNAs were used as controls.

### Cell Proliferation Assay

T24 or TRT-HU1 cells were plated onto 6-well culture plates at a density of 1×10^3^ cells per well in standard growth medium. When cells grew to ∼80% confluence, they were serum-starved for 16 h followed by incubation in serum-free medium containing 1 µM *as-*APF or inactive nonglycosylated control peptide for an additional 3 days. Cell proliferation was quantified by crystal violet staining. Briefly, cells were stained with crystal violet solution and quantified by dissolving stained cells in 10% acetic acid solution. Colorimetric reading was done by absorbance measurement at 570 nm.

### Western Blot Analysis

Whole cell lysates were prepared as described [53]. Proteins were separated by SDS-PAGE and transferred onto nitrocellulose membranes for immunoblotting analysis. ImageJ (v1.410, rsbweb.nih.gov/ij) was used for densitometry, which was followed by normalization against corresponding loading control bands.

### Immunofluorescence Microscopy

Cells were fixed with 4% paraformaldehyde and incubated with 1∶500 primary antibodies against p53, MDM2, USP2a or cyclin D1 (diluted 1∶500 in PBST) prior to incubation with 1∶250 Cy3.5-conjugated secondary antibody (red). Slides were mounted in Vectashield with DAPI and observed under a Zeiss microscope (Burlingame, CA). For BrdU staining, cells were incubated in labeling medium for 1 h before fixation and staining with anti-BrdU antibody according to the manufacturer’s instructions (Roche, Penzberg, Germany).

### Promoter Luciferase Assay

T24 cells were transiently transfected with a cyclin D1 promoter-luciferase construct, which was kindly provided by Dr. Osamu Tetsu at University of California, San Francisco [54] and varying concentration of USP2a^WT^. Two days after transfection luciferase activity was measured in whole cell lysates. As described in the manufacturer’s protocol, firefly and Renilla luciferase activities were measured with the Promega Dual-Luciferase reporter system (Promega, Madison, WI). Total protein was used for normalization. All experiments were carried out in triplicate and repeated 3 times using different preparations of plasmids.

### Statistical Analysis

The mean of 3 or more replicates was used as average. Data is presented as mean ± SD. The p-values were calculated using a standard unpaired Student’s *t*-test for simple comparisons. Statistical significance is displayed as p<0.05 (one asterisk) or p<0.005 (two asterisks).
